# Change and Monotony in the Factory

**Published:** 1924-06

**Authors:** 


### CHANGE AND MONOTONY IN THE FACTORY.

### THEIR EFFECT ON THE HUMAN MACHINE.

In the most recent of an interesting series of reports issued by the Medical Research Council, the Industrial Fatigue Research Board deals with the effects on the worker and his output of the repetition of operations on which he is engaged all day and every day.

### The Division of Labour.

Some idea of the extent to which the division of labour has been carried may be gathered from a recent statement that in a modern boot factory 350 separate processes are involved in making a pair of boots and placing them on the market. In the Report now presented the results are given of the systematic study of the extent and the effect on the worker of repetitive processes in a number of industries. Observations were made at three boot and shoe factories, two tin canister factories, a chain factory, a handkerchief factory, a soap factory, and other establishments. Note was made of the times spent on the chief manual or machine operation, on subsidiary operations, on the respective times spent in sitting or standing, and the duration of breaks due to walking about carrying material and other circumstances.

### Some Examples.

As an example of a truly monotonous form of occupation we may quote that known as cap padding in the tin canister industry, where girls insert a disc of linoleum or cardboard into tin caps at one second intervals all the day long. It is not surprising that the girls require a rest of five minutes every half-hour. As a type of operation in which there is variety the process known as lift cutting in the boot and shoe industry may be cited. It is performed by men who are seated, and it consists in stamping out, by means of steel cutters, the leather pieces which are afterwards built up into heels. The cuttings are repeated at intervals of 1 to 4 seconds. Each man has five different sized cutters at hand, and he constantly changes from one to another, so as to be able to cut out the leather with the minimum of waste; this requires some judgment on his part.

### The Effect of Change.

A second section of the Report deals more directly with the effect of change on the productive capacity of the worker. It embodies the results of a further series of investigations, both under general factory conditions and as the result of more intensive laboratory experiments. We are not surprised to read that where there is too frequent change there is a loss of efficiency when operatives who are just beginning to fall into the swing of the work have to stop and begin an entirely different process. On the other hand, it must surely be the merest truism to say that unvarying repetition work is conducive to fatigue and monotony, and that these almost neutralise the advantages of the rapidity and dexterity especially noticeable in the earlier part of the day and also, curiously enough, in the shape of final spurts when it is nearly time to go home.

### The Welfare of the Worker.

The real value of a report of this character lies in its suggestion to the individual employer of the various factors which by careful observation may be evaluated, and of the possibility of reducing the observations to some common form. The object of every good employer will be to secure efficiency so far as it is consistent with a proper regard for the well-being of the worker. The two things have, of course, intimate reactions on one another. Employers are more and more coming to realise that it pays as a matter of business to study the human machine and see that it is well cared for.

### Some of the Factors.

Some of the factors brought under review in the report are the intervals at which change of operation is desirable and economically possible; the effect of changes of posture; the cases in which it is desirable, and those in which it is not, for the worker to fetch his own material; the value of the dinner interval and of a short break for tea; the uses of rest and of conversation; and, not least, the temperament of the individual worker. A word of warning is given against relying too much on the opinions of operatives, either individually or in groups in regard to the question of working uninterruptedly at the same process or to that of changes of posture. Much depends on the way in which such questions are put to them, and they require some guidance on the subject.

### The Guidance of the Employer.

It is not merely the employee who needs guidance. The employer may, on first impulse, resent having suggestions made to him as to the way in which he should run his business; nor is it to be supposed for a moment that here and there will not be found an employer to whom all things such as are here referred to are matters of common usage. But this is certainly not true of the majority. If the investigators are properly informed, there have not been many attempts to introduce changes into repetitive work in industry.

### The Co-operation of Industries.

It is for these reasons that we say that the real value of such investigations and recorded observations as these lies in their suggestion to the captains of industry. The Industrial Research Board embodies in its membership many valuable types of experience. It is within its terms of reference not only to carry out schemes of research for promoting better knowledge of the relations of the conditions of employment to the functions of the human body, but also "to take steps to secure the co-operation of industries in the fullest practical application of the results of this research work to the needs of industry." This is the true test of the ultimate value of their work, and it would be interesting to learn what effective steps have been taken to this end.

